# A dosing strategy model of deep deterministic policy gradient algorithm for sepsis patients

**DOI:** 10.1186/s12911-023-02175-7

**Published:** 2023-05-04

**Authors:** Tianlai Lin, Xinjue Zhang, Jianbing Gong, Rundong Tan, Weiming Li, Lijun Wang, Yingxia Pan, Xiang Xu, Junhui Gao

**Affiliations:** 1 Department of Critical Care Medicine, Quanzhou First Hospital Affiliated to Fujian Medical University, Quanzhou, Fujian China; 2 Shanghai Nuanhe Brain Technology Co., Ltd, Shanghai, China; 3 Shanghai Biotecan Pharmaceuticals Co., Ltd, No. 180 Zhangheng Road, No LtdShanghai, China; 4 Beijing Center for Disease Prevention and Control, Beijing, China

**Keywords:** Clinician, Model, Reinforcement learning, Sepsis

## Abstract

**Background:**

A growing body of research suggests that the use of computerized decision support systems can better guide disease treatment and reduce the use of social and medical resources. Artificial intelligence (AI) technology is increasingly being used in medical decision-making systems to obtain optimal dosing combinations and improve the survival rate of sepsis patients. To meet the real-world requirements of medical applications and make the training model more robust, we replaced the core algorithm applied in an AI-based medical decision support system developed by research teams at the Massachusetts Institute of Technology (MIT) and IMPERIAL College London (ICL) with the deep deterministic policy gradient (DDPG) algorithm. The main objective of this study was to develop an AI-based medical decision-making system that makes decisions closer to those of professional human clinicians and effectively reduces the mortality rate of sepsis patients.

**Methods:**

We used the same public intensive care unit (ICU) dataset applied by the research teams at MIT and ICL, i.e., the Multiparameter Intelligent Monitoring in Intensive Care III (MIMIC-III) dataset, which contains information on the hospitalizations of 38,600 adult sepsis patients over the age of 15. We applied the DDPG algorithm as a strategy-based reinforcement learning approach to construct an AI-based medical decision-making system and analyzed the model results within a two-dimensional space to obtain the optimal dosing combination decision for sepsis patients.

**Results:**

The results show that when the clinician administered the exact same dose as that recommended by the AI model, the mortality of the patients reached the lowest rate at 11.59%. At the same time, according to the database, the baseline mortality rate of the patients was calculated as 15.7%. This indicates that the patient mortality rate when difference between the doses administered by clinicians and those determined by the AI model was zero was approximately 4.2% lower than the baseline patient mortality rate found in the dataset. The results also illustrate that when a clinician administered a different dose than that recommended by the AI model, the patient mortality rate increased, and the greater the difference in dose, the higher the patient mortality rate. Furthermore, compared with the medical decision-making system based on the Deep-Q Learning Network (DQN) algorithm developed by the research teams at MIT and ICL, the optimal dosing combination recommended by our model is closer to that given by professional clinicians. Specifically, the number of patient samples administered by clinicians with the exact same dose recommended by our AI model increased by 142.3% compared with the model based on the DQN algorithm, with a reduction in the patient mortality rate of 2.58%.

**Conclusions:**

The treatment plan generated by our medical decision-making system based on the DDPG algorithm is closer to that of a professional human clinician with a lower mortality rate in hospitalized sepsis patients, which can better help human clinicians deal with complex conditional changes in sepsis patients in an ICU. Our proposed AI-based medical decision-making system has the potential to provide the best reference dosing combinations for additional drugs.

## Introduction

Sepsis is a type of systemic inflammatory syndrome (SIRS) caused by the invasion of pathogenic microorganisms such as bacteria into the body. Sepsis and subsequent inflammatory responses can lead to multiple organ dysfunction syndrome (MODS) and even death if not treated promptly and accurately [1, 2].

The rate of sepsis incidence is high. In 2017, an estimated 48.9 million cases of sepsis were registered, and approximately 11.0 million sepsis-related deaths were reported worldwide, representing approximately 19.7% of all deaths globally [3]. At the same time, the treatment of sepsis requires many social and medical resources, posing a threat to personal physical and mental health and seriously affecting the quality of life of patients and their families [4–6].

Intravenous (IV) fluids and vasopressors (VPs) are commonly used to treat sepsis [7]. Most dosing combinations for sepsis patients focus on IV fluids and VPs because they are the most important elements in sepsis treatment; however, there remains no consensus on when and what amounts of each drug should be administered to sepsis patients [8, 9].

To address this problem, in late 2018, research teams at the Massachusetts Institute of Technology (MIT) and IMPERIAL College London (ICL) developed a medical decision-making system based on the deep-Q learning network (DQN) algorithm for sepsis treatment [10–12].

This was an innovative and pioneering system in the application of reinforcement learning techniques in the field of medicine dosing [12, 13]. Patients with sepsis require continuous IV and VP injections to maintain their blood pressure; however, the optimal dosing combination of IV fluids and VPs remains controversial [14]. An AI-based medical decision-making system extracts and learns information from a large number of clinical data and outputs the optimal therapeutic strategy by analyzing the outcomes of multiple treatment decisions [15]. The system outperformed human clinicians in determining the optimal dosing combination of IV fluids and VPs [16].

AI models, including reinforcement learning algorithms, are expected to provide patients with personalized treatment plans and improve their treatment outcomes [14, 15]. To better deal with the various complex clinical conditions of sepsis patients and obtain a more optimal treatment plan, we replaced the core algorithm used in the AI-based medical decision-making system developed by the research teams from MIT and ICL with the deep deterministic policy gradient (DDPG) algorithm [17]. The DDPG algorithm can handle high-dimensional input data and converges faster, making it better suited for medical data.

The medical decision-making systems proposed for sepsis patients have all been based on deep reinforcement learning algorithms, which have many advantages in terms of medical decision-making, such as the ability to handle sparse reward signals, making systems based on such algorithms adaptable to special patients, and allow a level of sensitivity in terms of different drug decisions [18–21]. Such a medical decision-making system can not only improve the survival rate of patients and reduce the pressure on social medical resources and family finances, it also helps human clinicians to make treatment decisions more effectively. Simultaneously, this system can provide a personalized treatment plan for each patient to optimize the outcomes of the complete individual treatment process [22–24].

## Methods

### Data

We used the same public intensive care unit (ICU) dataset applied by the research team at MIT and ICL, i.e., the Multiparameter Intelligent Monitoring in Intensive Care III (MIMIC-III) dataset [25, 26], which contains information on the hospitalizations of 38,600 adult sepsis patients over 15 years in age and meeting the internationally recognized sepsis 3 standard.

The data on 38,600 hospitalized patients over 15 years in age were first screened, and their vital signs within 72 h of contracting sepsis were extracted. The 72-h data were then divided into 4-h segments, and the data segments were aligned based on time. If multiple data points were found in a time segment, we calculated their average or sum according to the actual situation. For data segments with incomplete information, the K-nearest neighbor algorithm was used to estimate and fill in the fitted information to ensure that the data were as accurate as possible. We then removed the vital sign data that exceeded the clinical limits and normalized the data. A 48-dimensional feature vector was generated for each patient at each time step. Similar to the research teams from MIT and ICL, we used an auto-encoder method to expand the data features into 200 dimensions to improve the learning effect of the deep reinforcement learning model.

### Actions and rewards

As shown in Table 1, we divided the dosages of IV fluids and VPs into five integer dosing levels, where zero represents no addition of drugs, and the higher the level, the greater the quantity of drugs added. We then converted the IV fluid and VP dosing of each patient at each time point into the five dosing levels described above [27].

**Table 1:** Five levels corresponding to IV fluid and VP dosages

Dosing levels	0	1	2	3	4
Drug dosage					
IV (ml/h)	0	1–50	50–180	180–530	> 530
VP (µg/kg/min)	0	0.001–0.08	0.08–0.22	0.22–0.45	> 0.45

**Fig. 1:**
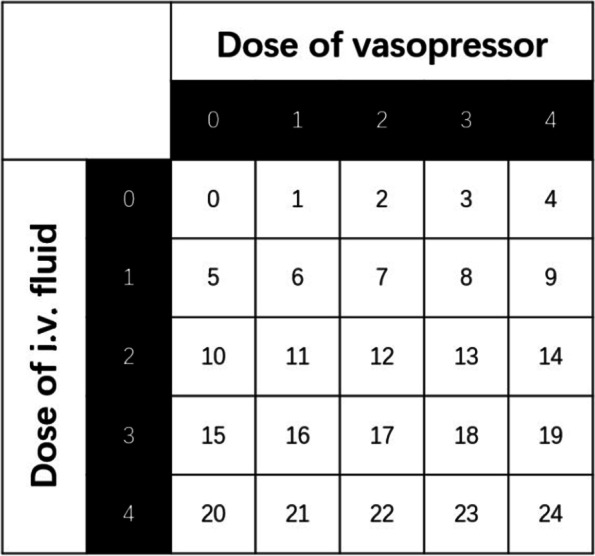
Each action corresponds to the combination of IV fluid and VP dosages

As shown in Fig. 1, the output of the medical decision-making system can be represented by a discretized tuple (IV dosing, VP dosing), resulting in a 5 × 5 action space, where each action corresponds to a tuple, that is, the combination of IV fluid and VP dosages [9].

The vital sign data of the patients will change with the dosing of IV fluids and VPs, and such a change determines the reward. The appropriate reward was calculated based on the Sequential Organ Failure Assessment (SOFA) score and lactate value, where the SOFA score represents the degree of organ failure and the lactate value measures the degree of cellular hypoxia in patients with sepsis [27]. The equation is as follows:

$$\mathrm{r}\left({s}_{t},{s}_{t+1}\right)={C}_{0}\left({s}_{t+1}^{SOFA}={s}_{t}^{SOFA}\&{s}_{t+1}^{SOFA}>0\right)+{C}_{1}\left({s}_{t+1}^{SOFA}-{s}_{t}^{SOFA}\right)+{C}_{2}tanh({s}_{t+1}^{Lactate}-{s}_{t}^{Lactate})$$

Here, C_0_ =  − 0.025, C_1_ =  − 0.125, and C_2_ =  − 2. The reward was negative when the SOFA score was higher. At the same time, when the SOFA score and lactic acid value increased, the reward was negative. If the patient eventually survived, the reward was increased by 15 points; otherwise, it was reduced by 15 points.

### Model architecture

1. Experience feedback

With respect to experience feedback, a weighted sampling method was used to set the initial probability of extracting data to the absolute value of the reward. The larger the reward is, the more significant the change in state, indicating that the input data are more conducive to model learning. If the state of the patient was discharge or death, the relevant values for the next state were set to zero.

2) Neural networks

A model based on the DDPG algorithm generally contains four neural networks, two online networks, and two target networks. Both online and target networks are subdivided into actor and critical networks. In our model, all four neural networks have two hidden layers and use the random batch gradient descent method and leaky RELU activation function. Meanwhile, critical networks apply equal advantages and value functions.

3) Algorithm flow

**Fig. 2:**
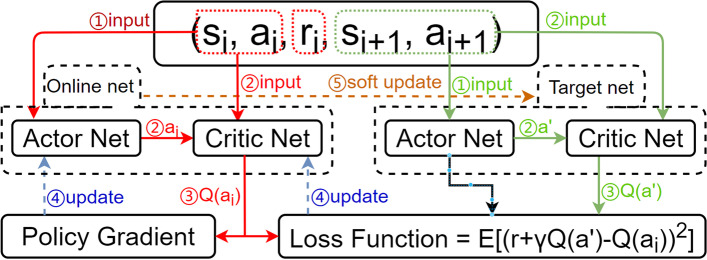
Structure of sepsis drug delivery algorithm

As shown in Fig. 2, the model first passes the samples drawn from the database to the actor network. Independent hot coding is used inside the network to obtain the coordinates of the action corresponding to each sample by changing the output form of the original 25 action probabilities to the probability of a specific action. We then use the original randomly selected action intelligence to select only the specified action and obtain the weight parameters of the actor network.

The actions produced by the two actor networks, together with the corresponding next state in the sample, are then passed to two critical networks. In other words, critical networks evaluate the actions produced by the actor network.

The loss function is then calculated using the Q data generated by the two critical networks, which in turn optimizes and updates the parameters of the critical online network.

Finally, the Q value produced by the critical online network is passed to the actor online network, and its policy gradient is updated. The parameters of the entire target network are then updated using soft updates. After many training cycles, the Q-value of the critic network is more accurately predicted, and the corresponding action of the actor network is improved.

4) Model architecture

We tested the performance of different reinforcement learning algorithms and corresponding parameter combinations on this data set. The algorithms include Double Q-learning, Dueling Networks, noise Nets, priority replay, and Multistep learning. Their corresponding parameters consist of exploring rate, learning rate, discount rate, number of neural network layers, etc. The algorithm and parameters with the lowest mortality were selected by the GridSearchCV method. The final selected model was an improved version of the classical DDPG algorithm. The main differences from the DDPG algorithm are as follows:

The connection allowing the agent to sample the environment is removed, and data are taken directly from the experience pool. Some random actions are also removed, and thus the agent chooses the same action from the experience pool. The action selected for the next state of each sample recorded in the experience pool is added.

## Results

**Fig. 3:**
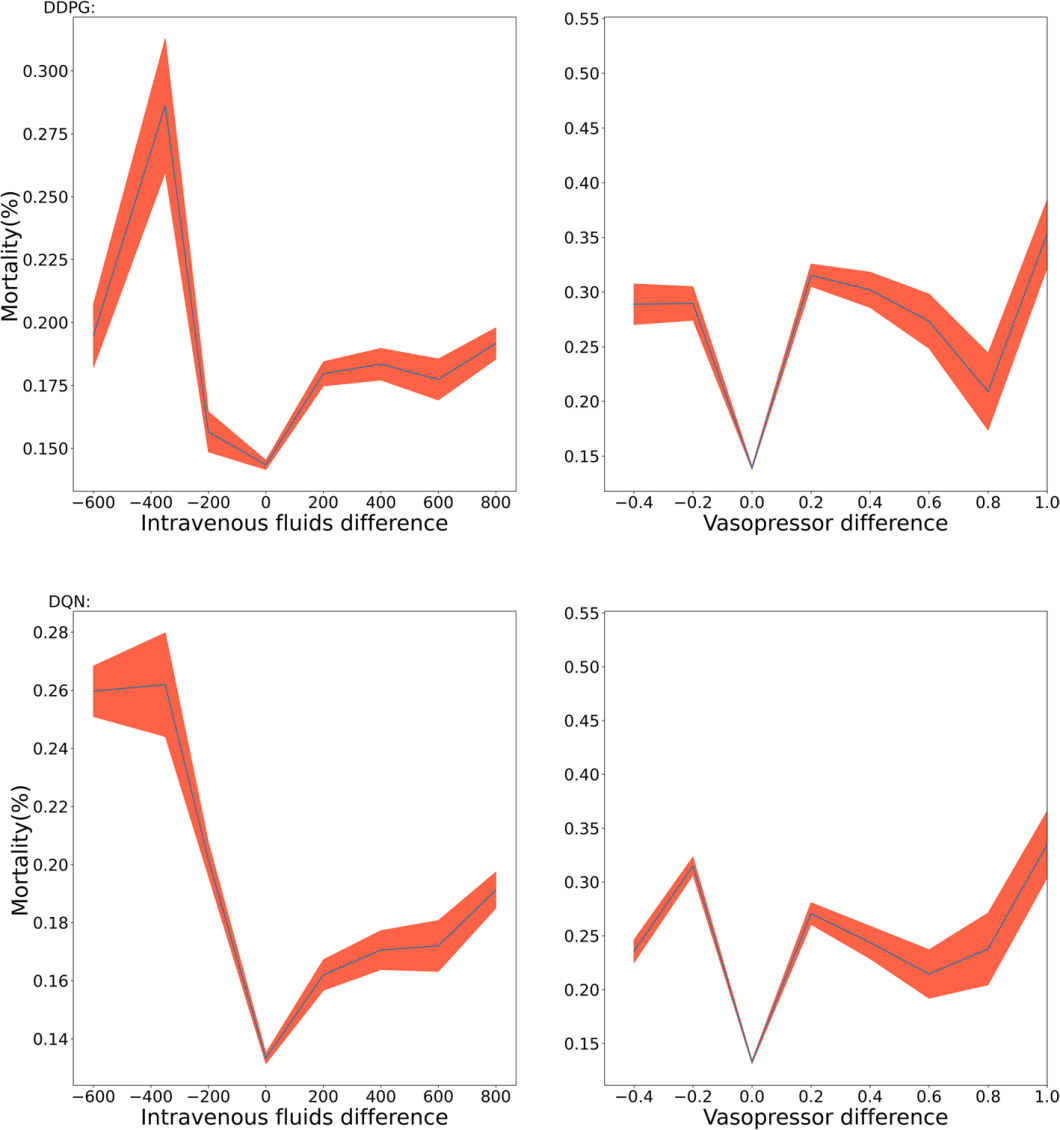
Mortality rate (y-axis) corresponding to the difference between the dosing given by the model and that given by the human clinician (x-axis). Differences in IV fluids and VPs affect the in-hospital mortality rate of sepsis patients. By analyzing the differences in the doses administered by the model and by human physicians at different time points, it can be seen that when the difference in dosing is zero, the mortality rate of the patients is the lowest

We adopted the U-curve method used by Raghu et al. [28] and the results are shown in Fig. 3. The U-curve method is a statistical method for evaluating clinical decision making by comparing the actions of a clinician with an evaluation policy, and measuring the associated outcomes. The idea behind the method is that a positive association between the difference between the clinician's policy and the evaluation policy and an outcome, such as mortality, suggests that the best outcomes occur when the clinician's actions align with the suggested actions. The U-curve is constructed by plotting the difference between the clinician's and evaluation policies against the outcome of interest, and the resulting shape of the curve represents the relationship between the policies and outcomes.

The upper part of Fig. 3 shows the change in the average mortality rate of hospitalized sepsis patients with the difference of dosing strategy between the DDPG model and the human clinicians. The left side shows the relationship between the mortality of patients and the difference between the IV fluid dosage given by the DDPG model and that given by a human clinician, which indicates that the patient has the highest survival rate when both treatment plans are the same. The right part shows the results of mortality by VP dosing difference, and the same conclusion can be drawn. The lower part of Fig. 3 shows the results of the DQN model. It can be seen that both models, DDPG and DQN, exhibit a typical ‘U’ shape, suggesting that the closer the human clinician's dosing strategy align with the suggested dosing strategy by models, the greater the survival rate of the patient.

The above results indicated the effectiveness of the dosing strategy given by the DDPG model. However, according to Gottesman et al. [29], such results may also be caused by confounding factors and the way actions were binned. Therefore, we further explore the effect of the two drug dosing combinations on the mortality rate of sepsis patients.

**Fig. 4:**
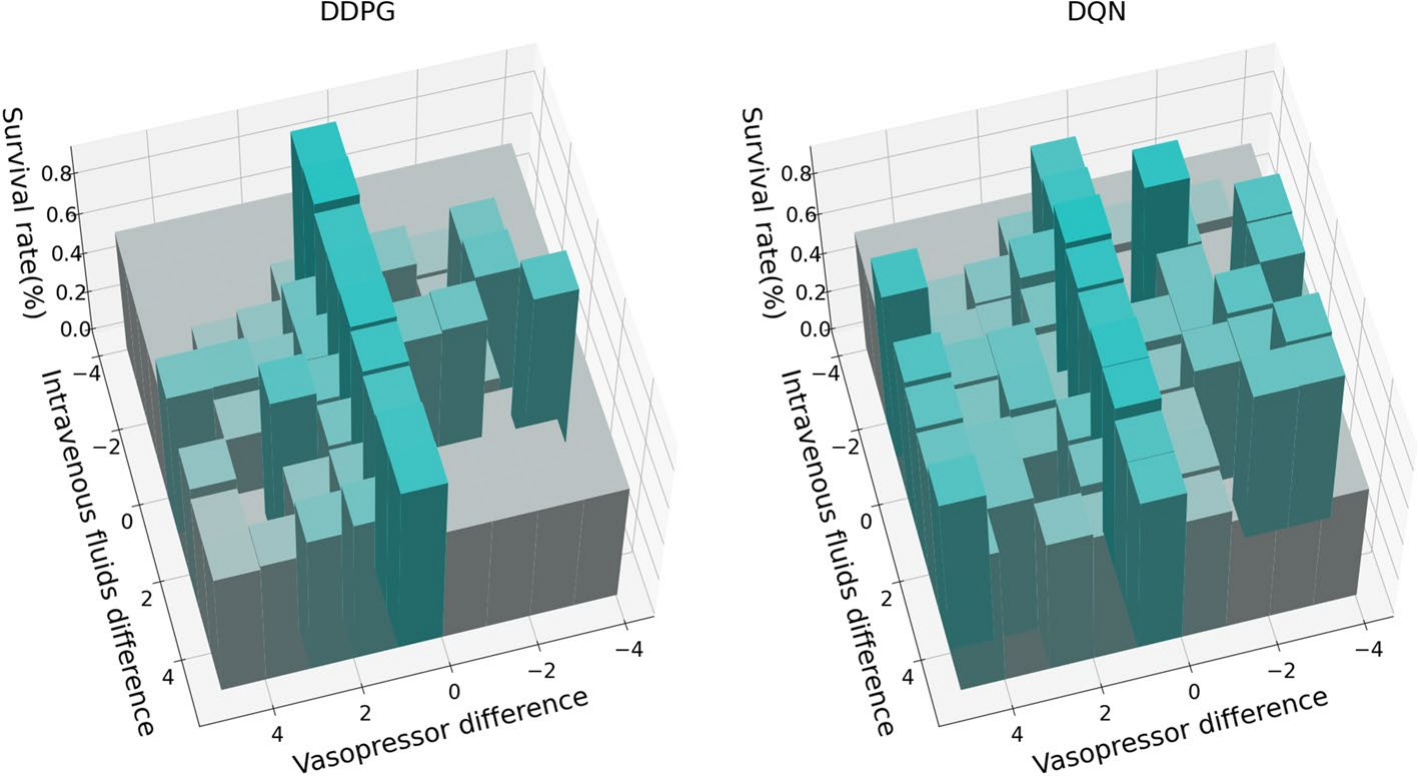
Relationship between in-hospital survival rate (z-axis) and differences in IV fluid (x-axis) and VP (y-axis) dosing administered by the models and human clinicians. The model is trained at a step size of 16.5 w, and the model outputs the drug dosing combination. If the number of results corresponding to the combination of drug doses is less than 50, such results will be removed because they are insufficient to explain the survival rate. The smaller the difference is between drug dosing given by the models and human clinicians, the better the survival rate of the sepsis patients. It can also be seen that the distribution map of the survival rate based on the DDPG algorithm is more concentrated than that based on the DQN algorithm. That is, the model based on the DDPG algorithm makes more treatment decisions similar to those of the human clinician than the model based on the DQN algorithm

As shown in Fig. 4, two three-dimensional histograms based on the DDPG and DQN algorithms were constructed to display the relationship between patient survival rate and the drug dosing combinations. The x-axis represents differences in IV fluids, and the y-axis represents differences in VP dosing, as administered by the models and human clinicians. The z-axis represents the survival rate of sepsis patients in an ICU. It can be seen that when the treatment strategies provided by human clinicians and models are more closely aligned, the patient's survival rate tends to be higher [30].

**Fig. 5:**
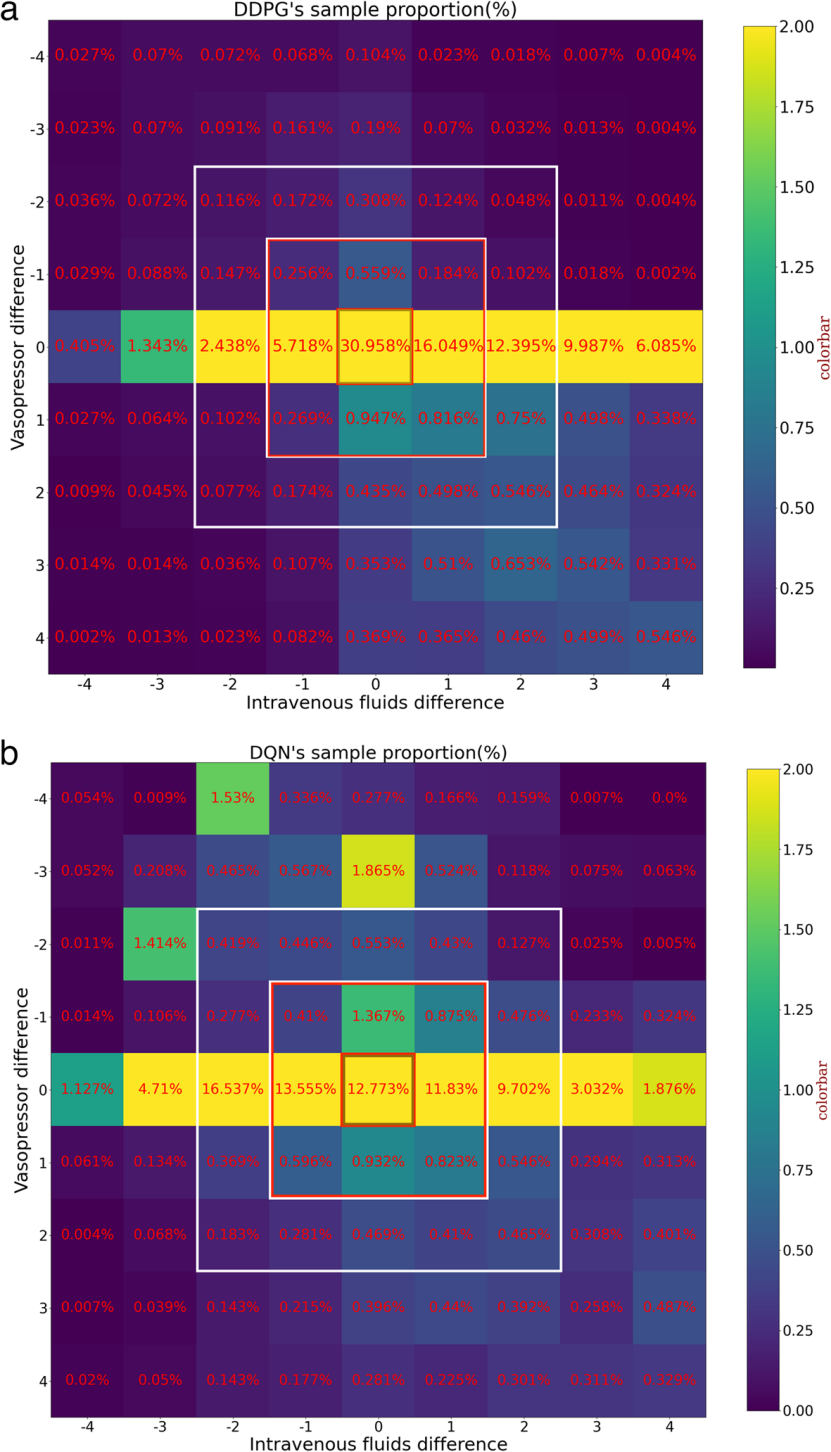
For differences in dosing combinations of IV fluids and VPs, each square represents the difference as a percentage of the total sample size. It can be seen that the difference in VP dosing is lower than that of IV fluids. It can be seen that the model is more inclined to make more drug dosing decisions. The model based on the DDPG algorithm recommended a sample size of 54.76% to receive more dosing (part a in Fig. 5), whereas the model based on the DQN algorithm recommended only 34.82% (part b in Fig. 5)

In order to further compare the results of the DDPG and DQN models, we drew heat maps for these two models, showing the relationship between patient survival and drug administration combinations, as shown in Fig. 5.

When the dose difference value was limited to within 2, as shown by the white box in Fig. 5, both the DDPG-based and DQN-based model generated about 42,000 sample sizes, accounting for 74.5% of the total. When the dosage of the clinician is exactly the same as the dosage recommended by the model, as shown in the brown box in Fig. 5, the number of samples obtained by the model based on the DDPG algorithm accounts for 30% of the total number of samples, which is 142.3% more than the one based on the DQN algorithm. With the gradual increase of the dose difference, the patient mortality rate obtained by the model based on the DDPG algorithm gradually increased, and the number of samples gradually decreased until the total number of samples was consistent with the model based on the DQN algorithm. It revealed that medical decisions generated by model based on DDPG algorithm tend to be more centralized and closer to those of human clinicians compared to DQN algorithm, meanwhile we also observed that mortality rate based on DDPG algorithm is smaller than that based on the DQN algorithm. At the same time, it was found that when the dose difference was zero, the patient's mortality rate was the lowest, and the greater the dose difference, the higher the patient's mortality rate.

**Fig. 6:**
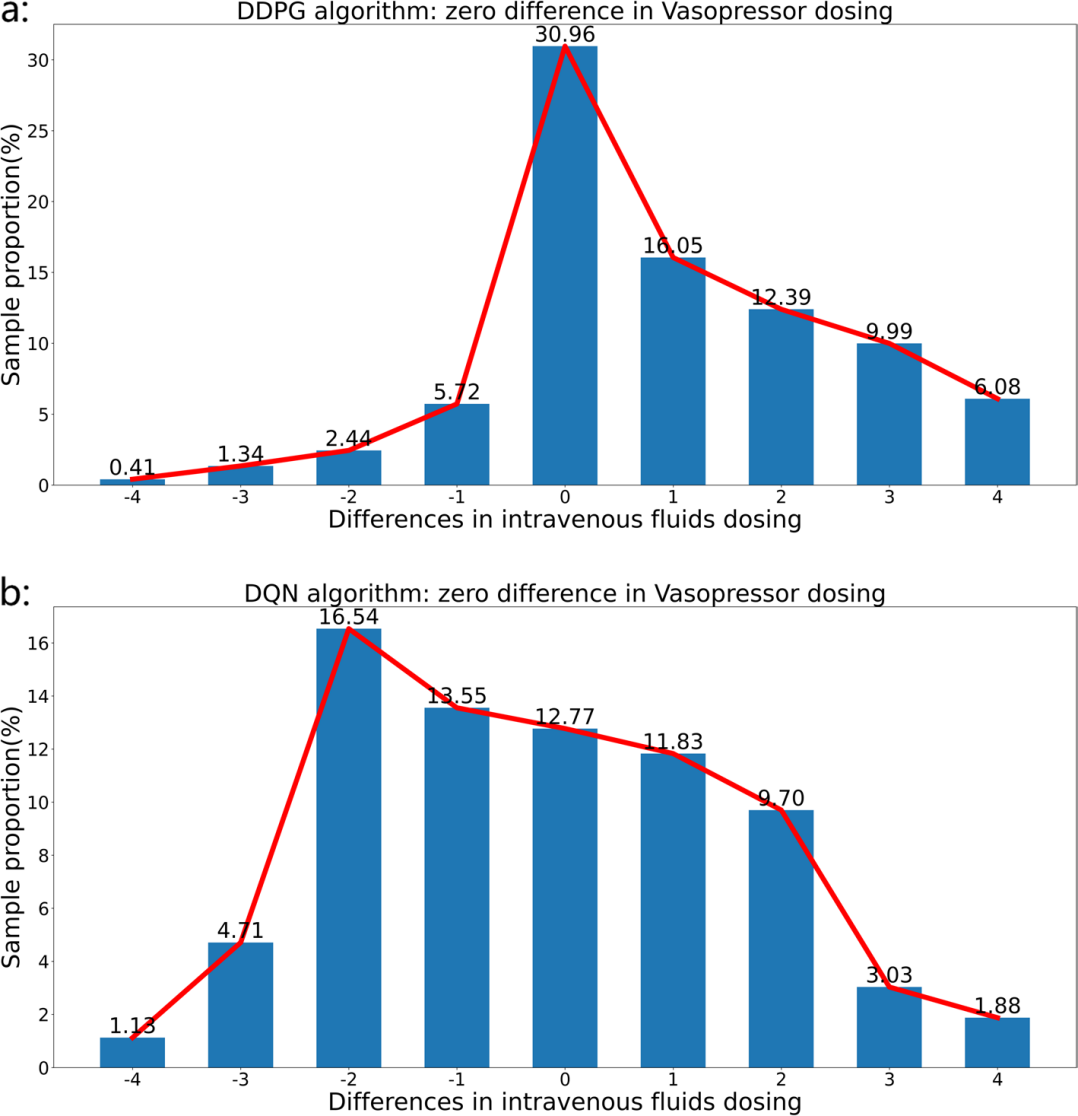
The differences in the sample size distribution of IV fluid dosage resulting from the use of the DDPG (part a in Fig. 6) and DQN (part b in Fig. 6) algorithms are plotted for a case in which the difference between the model and VP dosing decision of human clinicians is zero. As shown, the treatment plan generated by the model based on the DDPG algorithm is closer to the treatment plan generated by a human clinician

Specifically, when the difference between the VP doses administered by the model and those administered by a human clinician is zero, the sample size distribution of the differences in IV fluid dosage resulting from the DDPG and DQN algorithms is as shown in Fig. 6. As the figure indicates, the distribution of differences in IV fluid dosage resulting from the DDPG algorithm is more concentrated, which means that, compared with the DQN algorithm, the treatment plan generated by the model based on the DDPG algorithm is closer to the treatment plan generated by the human clinician.

Table 2 shows the sample size, proportion of samples, and mortality rate in different regions corresponding to the differences in dosing combination based on the use of the DDPG and DQN algorithms. It can be seen that model based on the DDPG algorithm produced more medical decisions that were closer to doctors than model based on the DQN algorithm, and at the same time DDPG-based model resulted lower mortality rates of patient compared to DQN-based model.

**Table 2:** Comparison of sample size, proportion of samples, and mortality rate in different regions corresponding to differences in dosing combination resulting from the use of the DDPG and DQN algorithms

Region	Brown		Red		White		Total	
Model	DDPG	DQN	DDPG	DQN	DDPG	DQN	DDPG	DQN
Sample size	17,293	7137	13,852	16,980	10,296	17,706	41,441	41,823
Sample proportion	30.96%	12.77%	24.8%	30.39%	18.43%	31.69%	74.19%	74.85%
Mortality rate	11.59%	14.17%	25.33%	25.94%	30.1%	25.27%	26.58%	25.58%

## Discussion

### Comparison of calculation efficiency

**Fig. 7:**
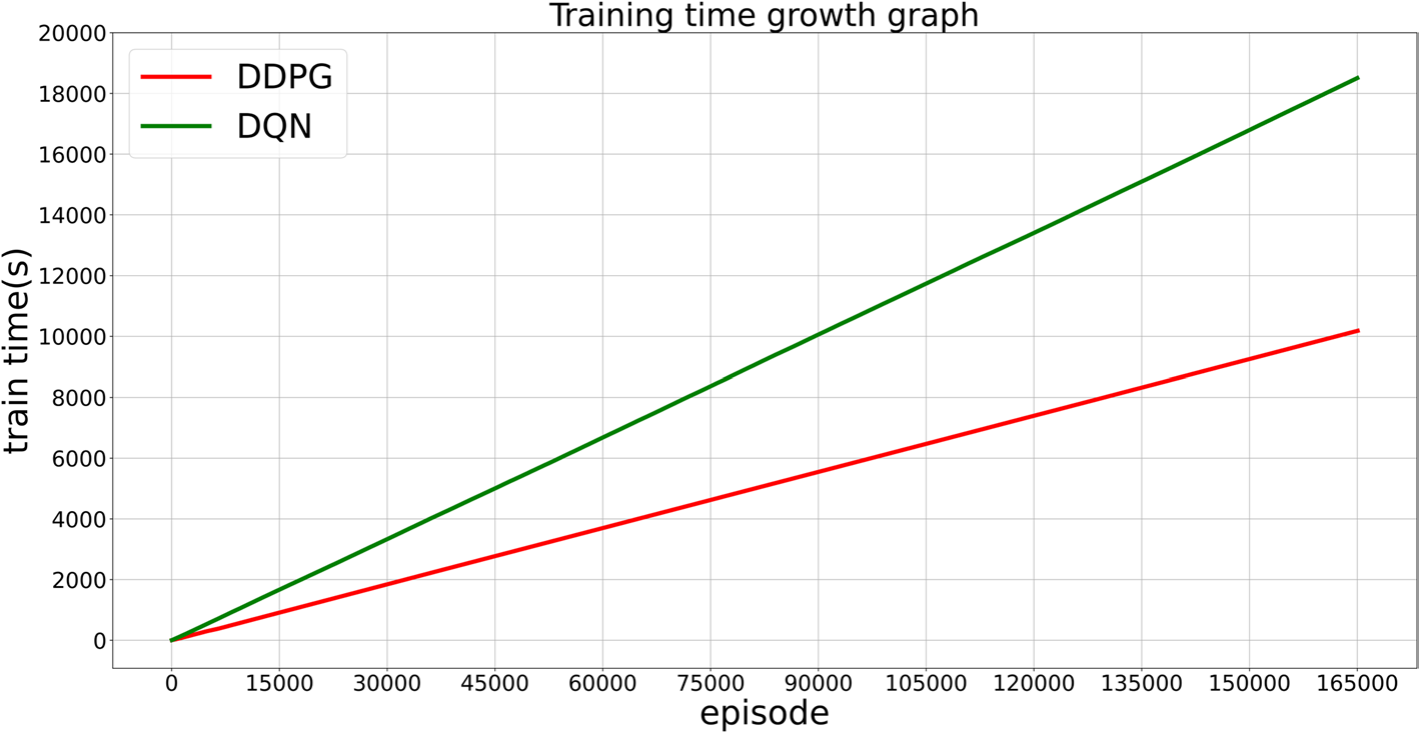
Relationship between the training time and training steps of the two models within the same environment. With the same number of steps and training under the same parameters and configurations, the time required by the model developed by the research teams from MIT and ICL was 1.7-times greater than that of our model

Patients in an ICU are frequently suffering from severe and rapidly deteriorating conditions. For patients in an ICU, time is of the essence. Using the same parameters and configurations as the model developed by the MIT and ICL research teams, we trained the AI clinicians to make decisions regarding drug dosing combinations for sepsis patients. The training efficiency of our model was drastically improved, as shown in Fig. 7, and a comparison of the efficiency becomes clearer when the number of data applied is larger. In particular, when a more precise treatment is required for the patient, the actions taken by the clinician can be larger than those taken by the IV and VP schemes. For the model developed by the MIT and ICL research teams, it may be difficult to train AI clinicians to apply multiple medical interventions.

### Model evaluation

**Fig. 8:**
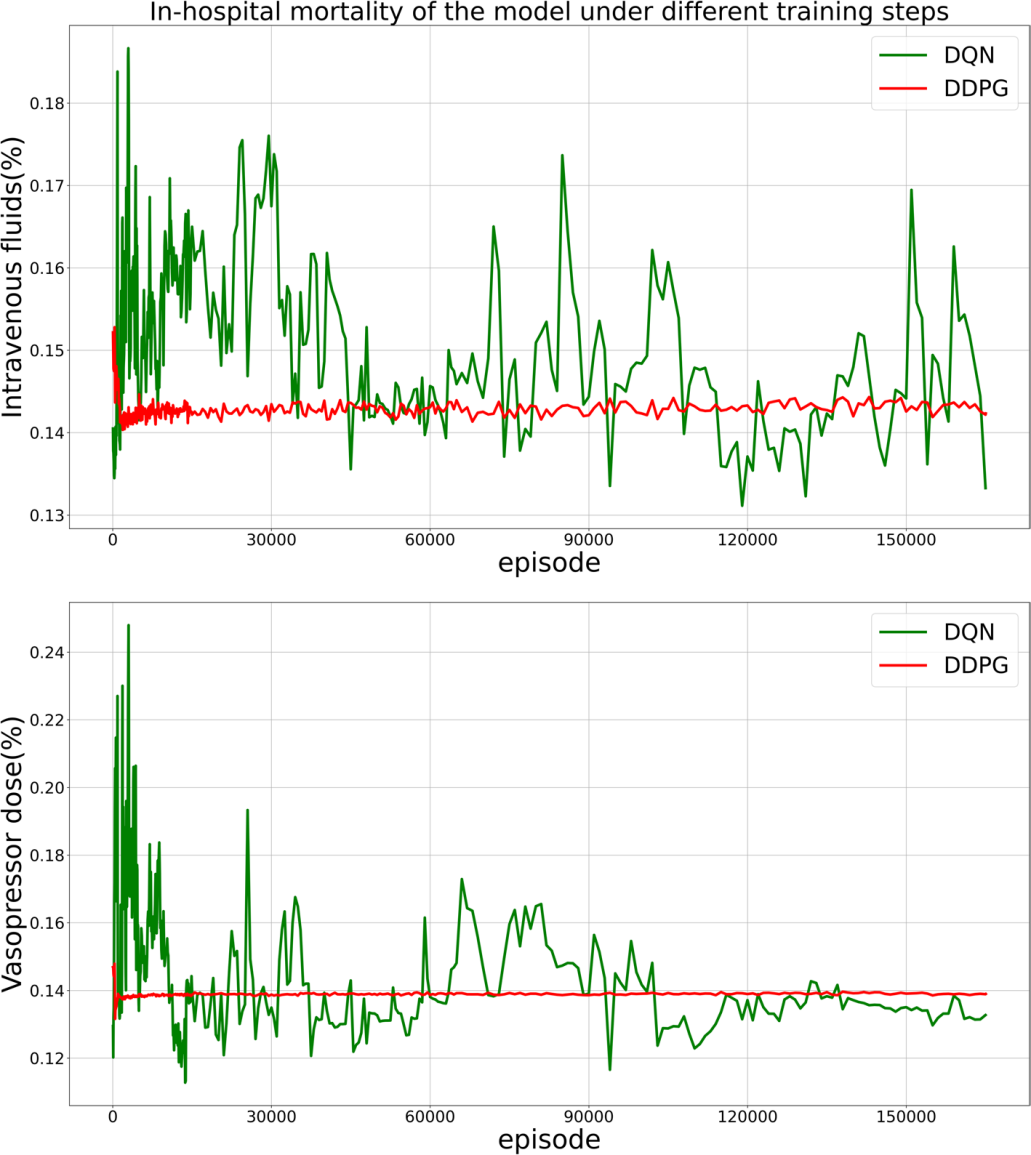
Two models with different training steps in the same training set predicted the in-hospital mortality rate after the test in the test set. In the first 5000 steps, 50 steps are used as the node to test the model. At this stage, we can see that the DDPG-based AI model has learned how to treat sepsis patients with drugs, whether intravenous fluids or vasopressors. The model developed by the research teams from MIT and ICL is still exploring the environment and only began to decline at 30,000 steps

As shown in Fig. 8, we took the parameters of the models trained using different steps from the continuous training and then tested these models against the test set to create a complete graph of hospital patient mortality through model training.

We analyzed the data on patients in an ICU, as shown in Fig. 8, and the patient mortality rate was 15.22% when we relied solely on the decision of the clinicians. When we added treatment by an AI clinician, the overall mortality rate decreased by approximately 1%. From the trend of the line graph, it appears that our AI clinicians are more stable than the AI clinicians developed by the MIT and ICL research teams and are better suited for use in an ICU. In conjunction with Figs. 7 and 8, we can see that our model converges at least 10-times faster than the model developed by the research teams from MIT and ICL.

**Fig. 9:**
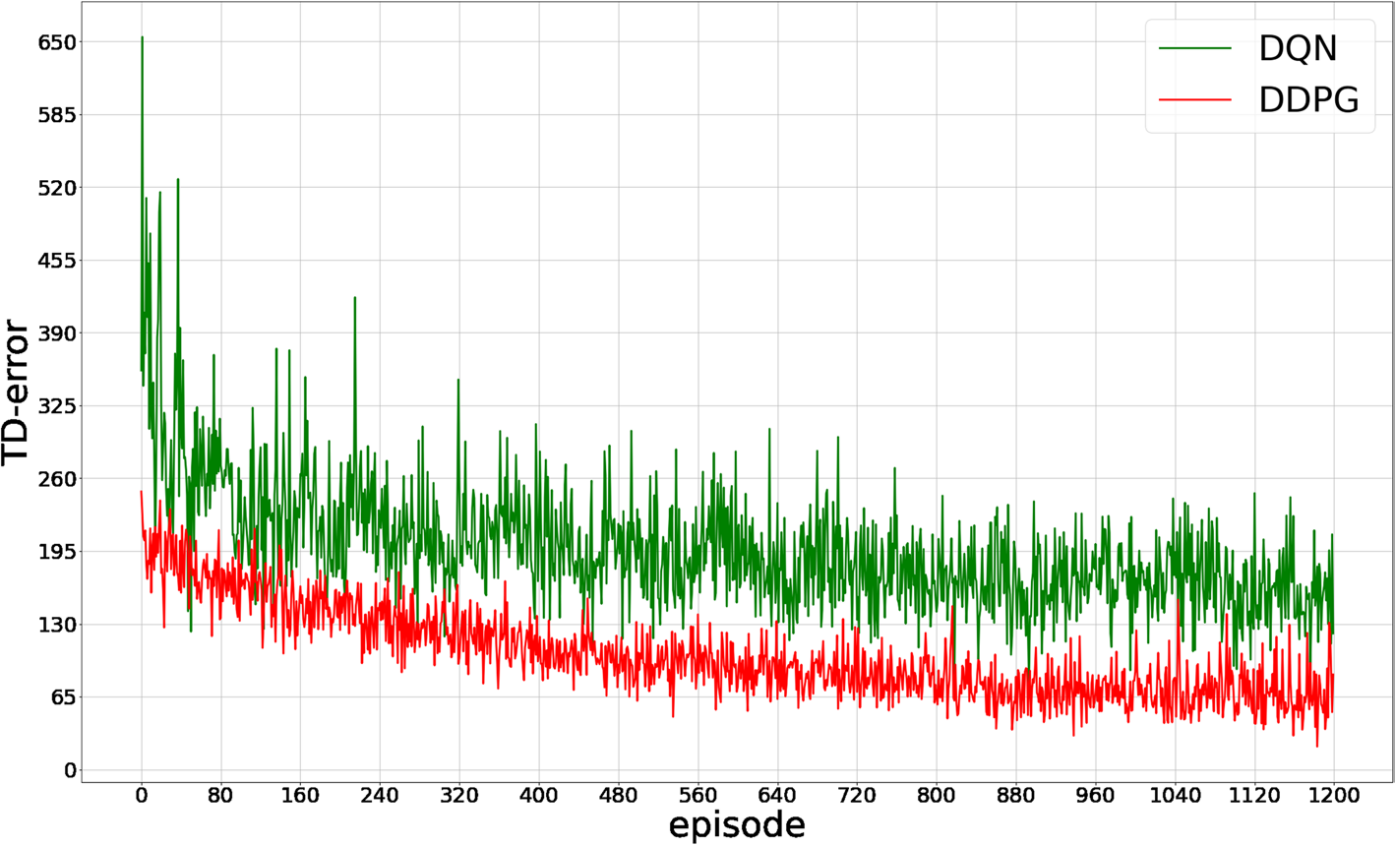
Change trend of TD-error after each training episode

As shown in Fig. 9, as the model training progressed, the TD error in the two models gradually decreased and became stable. The TD error of the DDPG model was consistently smaller than that of the DQN model throughout the training process.

### Further studies

As the research progresses, the model can be optimized in three additional ways:

1. Medical interventions lead to dynamic changes in the vital characteristics of the patients, preventing the model from steadily converging, and causing the recommended strategies to fluctuate within a small range in terms of the relationship between IV fluid volume and mortality.
2. To accurately control the administered dose, the output actions are not continuous, an issue that can be improved upon later.
3. It is difficult to optimize the hyperparameters of the model based on the actual environmental factors. In the future, we can adjust the hyperparameters to achieve a lower mortality rate.

## Conclusions

With the rapid development of big data and artificial intelligence technology, particularly in the medical field, the use of such technology is becoming increasingly mature. The application of AI-based technologies can help healthcare professionals not only to promptly detect clinical problems but also quickly formulate clinical treatment plans, which has a positive impact on improving the clinical service capability for critically ill patients [31].

Our AI decision-making system developed for sepsis clinicians can allow patient data to be shared with pre-trained AI clinicians, allowing the best treatment plan to be recommended to physicians. Clinicians can determine the final treatment plan by adding their subjective clinical judgment. We hope to apply this model to an ICU in the near future, improving the efficiency and quality of care, and find a treatment plan that is more appropriate for the patient.

## Data Availability

The datasets generated and analyzed as part of the current study are available at the MIMIC-III [32] repository (https://mimic.physionet.org/gettingstarted/access/); however, restrictions apply to the availability of these data, which were used under license for the current study, and thus are not publicly available. However, the data are available from the authors upon request and with permission from MIT Laboratory for Computational Physiology.

## Abbreviations

AI: Artificial Intelligence
DDPG: Deep Deterministic Policy Gradient
DQN: Deep Q-Learning Network
MIT: Massachusetts Institute of Technology
ICL: Imperial College London
ICU: Intensive Care Unit
IV: Intravenous injection
VP: Vasopressor
SOFA: Sequential Organ Failure Assessment
SIRS: Systemic inflammatory response Syndrome
